# Hepatitis E virus infection in the United States: Seroprevalence, risk factors and the influence of immunological assays

**DOI:** 10.1371/journal.pone.0272809

**Published:** 2022-08-05

**Authors:** María Belén Pisano, Christopher Campbell, Chimaobi Anugwom, Viviana Elizabeth Ré, José D. Debes

**Affiliations:** 1 Instituto de Virología “Dr. J. M. Vanella”, Facultad de Ciencias Médicas, Universidad Nacional de Córdoba, CONICET, Córdoba, Argentina; 2 Cancer Control Section, Minnesota Department of Health, St. Paul, MN, United States of America; 3 Division of Epidemiology and Community Health, University of Minnesota, Minneapolis, MN, United States of America; 4 Department of Medicine, Division of Infectious Diseases and International Medicine and Division of Gastroenterology and Hepatology, University of Minnesota, Minneapolis, MN, United States of America; CEA, FRANCE

## Abstract

In the United States (U.S.), a hepatitis E virus (HEV) seroprevalence between 6 and 21% has been described, with a decreasing trend. We aimed to investigate HEV infection in the U.S. population from 2009 to 2016, and examine the differences in seroprevalence using different assays. We used data from the National Health and Nutrition Examination Survey (NHANES-CDC) to estimate HEV seroprevalence and analyze demographic variables related to the infection. Additionally, we compared 4 serological tests used. The estimated HEV seroprevalence between 2009–2016 was 6.1% (95% CI: 5.6%-7.0%) for IgG and 1.02% (0.8%-1.2%) for IgM. Higher HEV IgG prevalences were found in older people, females, non-Hispanic Asians and those born outside of the U.S. The in-house immunoassay and the Wantai HEV-IgG ELISA presented the highest sensitivity values in the tested population. The highest specificity values corresponded to the DSI-EIA-ANTI-HEV-IgG assay. The kappa statistical values showed concordances no greater than 0.64 between the assays. HEV prevalence in our study was similar to previously reported, and a decline in the prevalence was observed through the NHANES assessments (from 1988 to 2016). The sensitivity and specificity of the assays varied widely, making comparisons difficult and highlighting the need to develop a gold standard assay.

## Introduction

Hepatitis E virus (HEV) is a non-enveloped virus with a positive stranded RNA genome of 7.2 kb and one of the most frequent causes of enterically transmitted acute viral hepatitis worldwide [1]. HEV is classified into 8 genotypes (1 to 8), many of them with animal reservoirs, of which 5 are known to infect humans.

This virus presents two different epidemiological scenarios: in tropical and sub-tropical regions, HEV genotypes 1 and 2 (HEV-1, HEV-2) are mainly transmitted via contaminated drinking water leading mostly to acute clinical or subclinical infection, while zoonotic transmission of HEV due to genotypes 3 and 4 (HEV-3, HEV-4) is present in resource-rich nations, mainly associated with pork meat as the main source of infection, and potentially leading to chronic infection in specific scenarios [2].

In the Americas, HEV-3 is endemic. While the majority of HEV-3 infections carry an asymptomatic course, a small minority (<5%) of cases develop acute symptomatic hepatitis E, and in immunosuppressed individuals, the infection can progress to chronic hepatitis [3].

HEV was first reported in the United States (U.S.) in 1997, but it has been present in the country for an unknown length of time [4]. Despite several studies having examined HEV in the country, neither its prevalence nor its impact is well-understood [5]. Moreover, a recent study from our group suggested that seropositivity for HEV could affect long-term liver physiology [6]. Two estimates of HEV seroprevalence from U.S. national studies exposed that HEV infection is not uncommon in the general population, with reported prevalences of 21% (Kuniholm et al. 2009) and 6% [5]. These studies were published based on serologic tests collected by the National Health and Nutrition Examination Survey (NHANES) in two periods of time: 1988–1994 and 2009–2016. In this regard, there appears to be a decline in the seroprevalence over the last 15 years with no concerted interventions directed at the disease. However, it is known that seroprevalence rates may be influenced by the variability of the assay used and by the sampling method with varying number of samples analysed, studied population, region of sample origin [2]. Many of the tests to assess HEV seroprevalence have not been well-characterized in the literature and divergent results have been documented [7–9]. Currently, there are several immunological assays available, all based on detection of antibodies (IgG and IgM) [10]. But there is no gold standard technique for HEV antibody detection, making it difficult to perform and accurate determination of the sensitivity or specificity of each test. Interestingly, different NHANES analyses have used different HEV tests raising the question of whether the perceived changes in HEV seroprevalence over time are due to external influences or to test differences. Additionally, demographic patterns have been studied in HEV infection and an association between age and HEV infection has been extensively shown [11, 12]. However, studies addressing other demographic characteristics including race/ethnicity, military service, education, and poverty have shown conflicting or insufficient evidence for correlation [5, 13, 14].

In this study, we provide a meticulous characterization of HEV infection in the United States from 2009 to 2016, examining the prevalence, short-term trends, and potential risk factors for HEV in the U.S. population. Additionally, we examine the differences in HEV seroprevalence from 1988–1994 using different assays, comparing the results with the period 2009–2016 and addressing the agreement between assays.

## Materials and methods

### Population

Data analysed during this work was obtained from the population included in the National Health and Nutrition Examination Survey (NHANES), conducted by the National Center for Health Statistics branch of the U.S. Center for Disease Control and Prevention (CDC). One of priorities of NHANES study (active since the 1960’s) is to create a nationally representative sample, providing weights to simulate the U.S. population based on their samples. Hence, the data collected by the NHANES is representative of the entire non-institutionalized, civilian, resident U.S. population [15]. In 1999, NHANES began continuously collecting data, and in 2009 this entity began serological testing of participants for hepatitis E. During this study we only examined data from years that included hepatitis E serological testing: 1988–1994 (NHANES III) and 2009–2016 (continuous NHANES). The NHANES III sera was analyzed with an in-house immunoassay, the DS-EIA-ANTI-HEV-G/M assay, the WANTAI IgG assay and the Western blot (this is publically available data) [16]. The recent NHANES cycles performed two types of serologic testing for hepatitis E: DS-EIA-ANTI-HEV-M–an IgM assay–and DS-EIA-ANTI-HEV-G–an IgG assay [9]. Both the continuous NHANES and NHANES III are cross-sectional studies that have been used to calculate national statistics.

Demographic variables included are age, ethnicity, birthplace, years lived in the United States (for immigrants), level of education and poverty level, as well as if they were civilian or military personnel. We also collated data on water source, shellfish consumption and history of blood transfusions.

### Seroprevalence estimation

Two methods of analysis to determine HEV seroprevalence in the NHANES III data were utilized: the first was to assume the survey weights were still valid and used code standard for complex survey design (method A); the second was to disregard the survey weights because they may no longer were applicable to the subset of sera used in the assays and instead use standardization (method B). Indeed, the svydesign function from a survey library in R was utilized to process the survey design data via applying weights and accounting for stratification and clustering. Later, Prevalence estimates were done using the svyciprop function with the output of the svydesign function as one of the parameters.

In the case of continuous NHANES, demographic analysis using HEV IgG seropositivity as the outcome variable was reported as the estimated percent of population seropositive with a 95% confidence interval. Transmission pathway analysis was done using a multivariate model to account for potential confounding factors and better assess the potential transmission pathway variables. This multivariate model contained race/ethnicity and being born in the U.S. as additional potential confounders.

### Serological tests evaluated

We evaluated the four different serological tests used to evaluate NHANES III and continuous NHANES sera for HEV [17]: 1) the NHANES in-house immunoassay, 2) the DSI-EIA-ANTI-HEV-IgG assay (DSI, Italy), 3) the Wantai HEV-IgG ELISA (WANTAI, China), and 4) an anti-HEV Western Blot (in house). In all the cases the sera were declared either positive or negative with no indeterminate classification [16–18].

#### Test comparison

We compared the performance of the assays on the same individuals in order to evaluate the HEV seroprevalence in U.S. Because all assays used nested subsets of the same NHANES III population (i.e. all persons tested with the Wantai assay were also tested by the in-house assay), we could directly compare the tests.

To determine the inter-assay reliability, we used the CompareTests function from the CompareTests library in R. The DS-EIA-ANTI-HEV-IgM assay is the only test that examines short-term exposure so was not included in the analysis. Using CompareTests, a kappa coefficient (ĸ) was calculated to determine how similarly the tests performed on a 1–1 basis. A ĸ of 0.8 or greater was considered a strong agreement [19]. In addition, we calculated the sensitivity and specificity of the four assays assuming each other one is the gold standard, since there is no established gold standard technique. So, we can say if a test produces on average more positive results than another test but not whether the test is more correct.

### Statistical analysis

In order to compare the performance implications of using an assay, two sets of analysis were done: 1) evaluate the seroprevalence from 1988–1994 (data from NHANES III) under the different assays and compare with the estimated seroprevalence in the period 2009–2016 (using continuous NHANES), 2) compare the assays used by calculating an agreement statistic. Data analysis was carried out using R 3.3.2 with the Hmisc, dplyr, survey, epitools and CompareTests libraries [20]. All four IgG assays done in NHANES III were included; however, the Wantai assay and the Western blot were conducted on a non-random subset of the data, so they did not produce accurate seroprevalence estimates.

The Svydesign function from the survey library was utilized to process the survey design data via applying weights and accounting for stratification and clustering. Prevalence estimates were done using the Svyciprop function. Demographic analysis using HEV IgG seropositivity as the outcome variable was reported as the estimated percent of population seropositive with a 95% confidence interval. Transmission pathway analysis was done using a multivariate model to account for potential confounding factors and better assess the potential transmission pathway variables. This multivariate model contained race/ethnicity and being born in the US as additional potential confounders.

## Results

### HEV seroprevalence and risk factors in the US 2009–2016

Table 1 shows the seroprevalence of HEV using survey weights (method A) and standardization (method B). For the estimates derived from random subsets of the NHANES III sera, the estimates using the survey weight method (A) and the standardization method (B) were similar. Using non-random subsets, estimates did differ significantly between both methods (changed by ~10%). In the United States, from 2009 to 2016, the estimated average HEV seroprevalence was 6.1% (95% CI: 5.6%-7.0%). In 2009–2010, the seroprevalence was 6.00% (5.1%-7.0%) and remained stable in 2011–2012 at 5.80% (4.6%-7.0%). Interestingly, HEV seroprevalence slightly declined to 4.6% (3.7%-6.0%) in 2013–2014 to later double to 8.10% (7.0%-10%) in 2015–2016 (S1 Table). The seroprevalence for the HEV IgM in the US varied between 0.5–1.6% each year between 2009–2015 (S1 Table).

**Table 1:** HEV seroprevalence using survey weights (method A) and standardization (method B).

	NHANES III		NHANES 2009–2016	
	Survey weights (method A)	Standarization (method B)	Survey weights (method A)	Standarization (method B)
In House EIA	0.208 (0.199, 0.22)	0.209 (0.201, 0.217)	NA	NA
DS-EIA-ANTI-HEV-G	0.165 (0.150,0.18)	0.162 (0.150, 0.174)	0.061 (0.056, 0.07)	0.058 (0.055, 0.061)
WANTAI HEV-IgG ELISA^a^	0.734 (0.699,0.77)	0.629 (0.586, 0.677)	NA	NA
Anti-HEV Western Blot^a^	0.683 (0.647,0.72)	0.588 (0.546, 0.636)	NA	NA

a-These were done on a non-random subset of DSI and hence should not be considered reliable estimates of national seroprevalence.

NA: not applicable

Table 2 describes a detailed categorization of HEV IgG seroprevalence in the period 2009–2016 by demographic characteristics. The HEV IgG trend in this period increased with age and was slightly higher in females. Non-Hispanic Asians had a significantly higher HEV seroprevalence (12.8%) than any other ethnicity. Those born outside of the U.S. had a higher HEV seroprevalence than those born inside the country (9.4% vs 5.5%). Conversely, among patients born outside the U.S., individuals who spent 5–10 years in the country were at an increased risk for positive HEV IgG compared to those that spent >30 years (RR 1.65; 95% CI: 1.25–2.2) (S2 Table).

**Table 2:** Seroprevalence of HEV IgG in demographic groups in U.S. in continuous NHANES (2009–2016).

Variable	Seroprevalence Estimate (%)	95% Confidence Interval
***Age in Years***		
Below 20	0.50	0.32–0.74
20–30	1.33	0.96–1.79
30–40	2.91	2.33–3.59
40–50	5.00	4.05–6.09
50–60	8.08	6.45–9.96
60–70	14.42	12.22–16.84
70–80	17.08	17.08–17.08
Above 80	18.48	15.88–21.32
***Gender***		
Male	5.79	5.27–6.35
Female	6.44	5.69–7.25
***Education***		
High School Equivalent or above	7.12	6.44–7.85
Less than High School	8.90	7.57–10.37
***Poverty Index***		
Above Poverty Line	6.25	5.62–6.93
Below Poverty Line	4.71	3.81–5.75
***Race/Ethnicity*** *		
Non-Hispanic White	6.85	5.93–7.86
Non-Hispanic Black	3.44	2.92–4.03
Mexican American	4.37	3.54–5.33
Other Hispanic	2.77	1.97–3.79
Non-Hispanic Asian	12.82	11.13–14.67
Non-Hispanic Other	4.42	2.51–7.14
***Military Status***		
Is/Was in Military	6.21	4.89–8.04
Never in Military	6.46	5.78–7.19
***Birthplace***		
Inside U.S.	5.49	4.91–6.12
Outside of U.S.	9.37	8.14–10.72
***Years spent in U*.*S*.** **		
Less than 5	6.30	4.52–8.51
5–10	9.06	6.93–11.58
10–20	8.38	6.54–10.54
20–30	10.04	7.65–12.88
More than 30	12.46	9.81–15.51

*Race/Ethnicity estimate 2011–2016 only because Asian race missing in 2009–2010

**Years spent in U.S. only assessed in persons born outside of the U.S.

We found no significant difference in HEV seroprevalence between different education levels or income groups (S2 Table). Of all other demographic variables addressed, only military status was found to be significant, showing a protective effect (RR = 0.69, 95% CI: 0.53–0.90).

The univariate and multivariate analyses addressing the impact of known risk factors, showed no statistically significant associations. Birthplace outside the U.S. (RR = 1.75), race/ethnicity (Asian RR = 2.18, Hispanic RR~0.63) and age (RR = 1.05 per year) were, however, found to be statistically significantly associated with HEV infection (S2 Table, data not shown for age RR).

### Comparison and correlation of seroprevalence with different HEV tests used in NHANES

Relative sensitivities among HEV tests are displayed on Table 3.

**Table 3:** Comparative sensitivity of HEV assays from NHANES III.

	In House EIA	DS-EIA-ANTI-HEV-G	WANTAI HEV-IgG ELISA	Anti-HEV Western Blot
Gold Standard	Sens (95% CI)	Sens (95% CI)	Sens (95% CI)	Sens (95% CI)
In House EIA	**X**	**0.48 (0.47, 0.50)**	**0.86 (0.85, 0.87)**	**0.77 (0.75, 0.84)**
DS-EIA-ANTI-HEV-G	**0.96 (0.95, 0.97)**	**X**	**0.96 (0.95, 0.98)**	**0.97 (0.95, 0.98)**
WANTAI HEV-IgG ELISA	**0.97 (0.96, 0.98)**	**0.56 (0.53, 0.59)**	**X**	**0.86 (0.84, 0.87)**
Anti-HEV Western Blot	**0.96 (0.94, 0.97)**	**0.62 (0.59, 0.65)**	**0.94 (0.93, 0.95)**	**X**

Using method A, the seroprevalence estimates in the NHANES III data by the in-house assay (20.8%) and the DS-EIA (16.5%) showed statistically significant difference from one another (Table 1). The Wantai and Western blot tests were performed on a non-random subset of the sera tested by the DS-EIA kit and hence could not be used to infer the true U.S. seroprevalence.

For the estimates derived from random subsets of the NHANES III sera, the estimates using the survey weight method (A) and the standardization method (B) were similar. Using non-random subsets, estimates did differ significantly between both methods (changed by ~10%).

The in-house assay had a high sensitivity when assuming any of the other assays were the gold standard, but other tests had a wide range of sensitivities when tested against it as the gold standard (48–86%). Conversely, the DS-EIA assay had consistently low sensitivity (48–62%) and other tests had a high sensitivity when compared against it (96–97%) (Table 3). When using any other assay as the gold standard, the in-house assay showed low specificity (38–79%), whereas the DS-EIA had a consistently high specificity (94–99%). The Wantai ELISA had variable specificity (42–88%), and the Western Blot had similarly variable specificity (54–85%) (Table 4).

**Table 4:** Comparative specificity of HEV assays from NHANES III.

	In House EIA	DS-EIA-ANTI-HEV-G	WANTAI HEV-IgG ELISA	Anti-HEV Western Blot
Gold Standard	Spec (95% CI)	Spec (95% CI)	Spec (95% CI)	Spec (95% CI)
In House EIA	**X**	**0.99 (0.98, 0.99)**	**0.88 (0.83, 0.91)**	**0.80 (0.76, 0.84)**
DS-EIA-ANTI-HEV-G	**0.79 (0.78, 0.80)**	**X**	**0.42 (0.40, 0.43)**	**0.54 (0.52, 0.56)**
WANTAI HEV-IgG ELISA	**0.54 (0.49, 0.58)**	**0.94 (0.92, 0.96)**	**X**	**0.85 (0.81, 0.88)**
Anti-HEV Western Blot	**0.38 (0.34, 0.42)**	**0.95 (0.93, 0.97)**	**0.66 (0.63, 0.69)**	**X**

Table 5 shows the Kappa agreement score. The DS-EIA and the Wantai had roughly similar agreement statistics when they were compared to the in-house assay (ĸ~0.55). The agreement statistic for the DS-EIA vs Wantai was weaker (ĸ~0.35). The strongest agreement statistic observed in the comparison of the Wantai ELISA with the Western Blot (ĸ~0.64)–still fell short of a strong ĸ value.

**Table 5:** Kappa agreement of HEV assays from NHANES III.

	In House EIA	DS-EIA-ANTI-HEV-G	WANTAI HEV-IgG ELISA	Anti-HEV Western Blot
	ĸ (95% CI)	ĸ (95% CI)	ĸ (95% CI)	ĸ (95% CI)
In House EIA	**X**	**0.54 (0.52, 0.56)**	**0.59 (0.54, 0.63)**	**0.39 (0.35, 0.44)**
DS-EIA-ANTI-HEV-G	**X**	**X**	**0.35 (0.33, 0.38)**	**0.48 (0.45, 0.51)**
WANTAI HEV-IgG ELISA	**X**	**X**	**X**	**0.64 (0.61, 0.68)**
Anti-HEV Western Blot	**X**	**X**	**X**	**X**

## Discussion

Previous studies have found a declining trend of HEV prevalence in the U.S. from 1988 to 2016, and in the following years in population-based cohorts (such us patients with liver injury) [21, 22]. Our study focused on a detailed analysis of HEV infection in the United States in the last period, and on addressing if the difference in the tests previously used had an impact in the HEV trend in the country.

The HEV prevalence obtained for the period 2009–2016 was similar to that found in the previous paper analyzing the continuous NHANES HEV serology [5]. The individual 2-year HEV seroprevalence cycles were also similar, with the highest HEV seroprevalence in 2015–2016 (8.08%) and the lowest seroprevalence in 2013–2014 (4.6%). In NAHNES III a subset of the population only was analysed for HEV, but we do not expect this to bias the findings as it was a random subset. The relatively constant HEV IgM seroprevalence obtained suggests that new HEV infections occur in individuals of all ages, consistently with prior findings [23]. Although it is not clear if transmission pathways vary by age group, a surveillance study noted that patients with infections acquired outside the U.S. were much younger on average compared to those who acquired them domestically [24]. In our study, we observed that non-Hispanic Asians (specific definition by the NHANES data) had a significantly higher average HEV seroprevalence compared to other ethnic groups. The reason for this finding is unclear. However, prior to 2011, non-Hispanic Asians were not included as a demographic group of interest; and it is possible that Asians born outside of the U.S. (shown to have a high seroprevalence of HEV), now included in subsequent NHANES, could be driving the higher seroprevalence noted in this racial group [25, 26]. In general, individuals born outside of the U.S. showed a higher HEV seroprevalence, suggesting foreign acquired cases of HEV could be a significant contributor to the overall national seroprevalence in the U.S. [23].

Surprisingly, we found a reduced risk association between military status and HEV infection. Military deployment to resource-limited regions has been associated with increased infection risk, and assignment to areas thought to have high HEV burden for long periods of time is expected to increase the risk of infection [27]. It is plausible for a diet-related causation to be considered as protective, as military personnel might adhere to a more restrictive diet [28]. In addition, it is quite conceivable that only a part of the cohort of deployed individuals were sent to resource limited or HEV high-risk areas. However, this association could be related to chance alone, and should be confirmed by other studies before further speculations.

In line with previous studies, we found no significant evidence of an association between HEV infection and the different transmission pathways such as unsafe water, shellfish, and blood-born transmission; in both the univariate and multivariate analyses [5, 13]. However, this does not completely disregard them as putative risk factors, as transient exposures to these transmission pathways cannot be accounted for. Moreover, temporal associations are not clearly apparent in population-based studies and, as discussed below, the questions about diet in NHANES were performed on 24h-recall. This should be interpreted with caution as prior studies have shown HEV seroprevalence associations with these variables, outside the U.S. [29, 30].

An issue to be considered for HEV epidemiological analyses, is the wide variability of the tests used to determine seroprevalence rates, which makes comparisons difficult. Unfortunately, the sensitivity and specificity of available assays vary widely; this may account for the discrepancies among published rates of anti-HEV antibody in various populations around the globe [31].

We observed that when using the DSI-EIA applied to NHANES III sera, the seroprevalence of HEV IgG (16.2%) presented a significant departure from the previous estimate using the in-house assay (20.9%) and trending towards, but still distant, from modern DSI-EIA estimates (6,1%). Based on the results of DSI-EIA prevalence, the HEV seroprevalence in U.S. showed a decline over time (from 16.2% to 6%). However, when analysing the in-house assay results, there is a wider difference between the estimates of seroprevalence in the two periods studied (from 20.9% to 6%), which illustrates the necessity of using the same assay, or at least tests with similar performances. The two most similar tests used were the Wantai ELISA and the Western Blot. The other tests were ELISAs, which may differ for multiple reasons, most notably by which part of the protein the test is detecting, but also what threshold of signal qualifies as a positive result [8]. These differences have previously been observed in other studies form other parts of the world, where discordant serological results have been reported [2, 31].

To our knowledge, this is the first seroepidemiologic study to examine differences between commercial HEV kits in the United States using NHANES data. In previous studies, the population has either been clinical subjects, suspected to have HEV [10, 32]; or a mix of blood donors and people suspected to have been infected with HEV [9]. This study examines a general population without consideration of risk for HEV infections and examines the differences between the commercial assays. The general population has a far lower seroprevalence than people suspected to have HEV, as such the decrease in quality of the agreement statistic between this study is likely due to the test being designed as a clinical test, not an epidemiological screening tool. Overall, of the evaluated HEV tests, Wantai appeared to have the highest sensitivity, which would make it a potential test for epidemiological studies as previously postulated [33], and the DS-EIA appeared to have the highest specificity, making it suitable for diagnosis.

Due to the retrospective nature of this study, it has several limitations. Although the additional data from years 2011–2016 improved our ability to detect smaller associations and temporal trends, the NHANES dataset did not substantially change, so structural limitations of NHANES still exist. 1)-The NHANES dataset includes two 24-hour food recalls. To enable in-depth risk factor analysis, extensive food recalls are required to allow for a thorough investigation of the highly suspected pathway of pork consumption. Furthermore, the prolonged incubation period of HEV could render 24-hour food recalls less helpful. 2)-Ease of data collection and de-identification may make collection of variables in a categorical format (such as in length of stay in the U.S) simpler for analysis but less accurate. Continuous variables in some cases may help determine more precisely if, for example, individuals with 5–10 year stay in the U.S. show a reduced risk for HEV infection. 3)-NHANES chooses a limited number of geographic regions per cycle, leading to a potential increased variability between cycles. 4)-While many of the demographic variables in the NHANES database are constant (such as race, birthplace, etc.), other variables such as water source, may change over time. The inability to assess the temporal relationships between variables may limit the ability to detect statistically significant associations. 5)-Only two of the major immunological assays were used during different NHANES testing, designed for clinical use, not epidemiologic surveys, which may contribute to an inflated seroprevalence estimate.

In summary, our study shows that the decline in HEV seroprevalence through the NHANES studies (from 1988 to 2016) was real but the wide range of the seroprevalence varied depending on the assay used. The variation in the kits for determination of HEV serology highlights a need to develop a gold standard assay or to standardize existing assays when conducting nationwide seroepidemiologic surveys.

## Supporting information

S1 Table Seroprevalence of HEV in the United States 2009–2016. (DOCX)

S2 Table Summary of individual models of HEV infection risk in U.S. (2009–2016) adjusted for age and gender. (DOCX)
