# Supplementary material for: Hepatitis E virus infection in the United States: Seroprevalence, risk factors and the influence of immunological assays
Source: PLoS One. 2022 Aug 5;17(8):e0272809. doi: 10.1371/journal.pone.0272809 (PMC9355204; doi:10.1371/journal.pone.0272809)
Supplement: S1 Table — (DOCX) [file pone.0272809.s001.docx]

**Supplemental table S1.** Seroprevalence of HEV in the United States 2009-2016.

| **NHANES Cycle** | **IgG Assay**  **Seroprevalence**  **(95% CI)** | **IgM Assay Seroprevalence**  **(95% CI)** |
| --- | --- | --- |
| 2009-2010 | 6.00% (5.1%, 7.0%) | 0.50% (0.3%, 0.8%) |
| 2011-2012 | 5.80% (4.6%, 7.0%) | 1.60% (1.2%, 2.2%) |
| 2013-2014 | 4.60% (3.7%, 6.0%) | 0.70% (0.3%, 1.1%) |
| 2015-2016 | 8.10% (7.0%, 10.0%) | 1.20% (0.9%, 2.2%) |
| Overall 2009-2016 | 6.10% (5.6%, 7.0%) | 1.00% (0.8%, 1.2%) |
