# Supplementary material for: Hepatitis E virus infection in the United States: Seroprevalence, risk factors and the influence of immunological assays
Source: PLoS One. 2022 Aug 5;17(8):e0272809. doi: 10.1371/journal.pone.0272809 (PMC9355204; doi:10.1371/journal.pone.0272809)
Supplement: S2 Table — (DOCX) [file pone.0272809.s002.docx]

**Supplemental table S2.** Summary of Individual Models of HEV infection Risk in U.S. (2009-2016) Adjusted for Age and Gender.

|  | **Risk Ratio Estimate** | | **95% CI** | | **p-value** |
| --- | --- | --- | --- | --- | --- |
| *Education Level* |  | |  | |  |
| High School or Above | 1.00 | | REF | |  |
| Lower than High School | 1.07 | | 0.91 – 1.25 | |  |
| *Poverty Index* |  | |  | |  |
| Above Poverty Level | 1.00 | | REF | |  |
| Below Poverty Line | 1.05 | | 0.88 – 1.25 | |  |
| *Race** |  | |  | |  |
| Non-Hispanic White | 1.00 | | REF | |  |
| Non-Hispanic Asian | 2.18 | | 1.85 – 2.57 | |  |
| Non-Hispanic Black | 0.68 | | 0.56 – 0.82 | |  |
| Mexican American | 1.16 | | 0.94 – 1.43 | |  |
| Other Hispanic | 0.63 | | 0.46 – 0.87 | |  |
| Other | 0.95 | | 0.65 – 1.40 | |  |
| *Military Status* |  | |  | |  |
| No | 1.00 | | REF | |  |
| Yes | 0.69 | | 0.53 – 0.90 | |  |
| *Birthplace* |  | |  | |  |
| Born in U.S. | 1.00 | | REF | |  |
| Born outside of U.S. | 1.75 | | 1.49 – 2.06 | |  |
| *Years in U.S.*** |  | |  | |  |
| More than 30 | 1.00 | | REF | |  |
| Less than 5 | 1.22 | | 0.84 – 1.76 | |  |
| 5-10 | 1.65 | | 1.25 – 2.22 | |  |
| 10-20 | 1.29 | | 0.98 – 1.69 | |  |
| 20-30 | 1.22 | | 0.93 – 1.60 | |  |
| *Ever Blood Transfusion* |  | |  | |  |
| No | 1 | | REF | |  |
| Yes | 0.95 | | 0.82 – 1.09 | |  |
| *Recent Shellfish* |  | |  | |  |
| No | 1.00 | | REF | |  |
| Yes | 1.10 | | 0.96 – 1.25 | |  |
| *Tap Water Source* | |  | |  | |
| Community Supply | 1.00 | | REF | |  |
| Don't Drink Tap | 1.05 | | 0.53 – 2.08 | |  |
| Well or rain cistern | 1.07 | | 0.79 – 1.46 | |  |
| Spring | 1.74 | | 0.88 – 3.46 | |  |
| *Race/Ethnicity Analysis with 2011-2016 data due to Asian race classification missing in 2009-2010  **Years spent in U.S. only assessed in persons born outside of the U.S. | | | | | |
